# Pregnancy and Neonatal Outcomes in Maturity-Onset Diabetes of the Young: A Systematic Review

**DOI:** 10.3390/ijms26136057

**Published:** 2025-06-24

**Authors:** Franciszek Ługowski, Julia Babińska, Katarzyna Makowska, Artur Ludwin, Paweł Jan Stanirowski

**Affiliations:** 1st Department of Obstetrics and Gynecology, Medical University of Warsaw, 02-091 Warsaw, Poland

**Keywords:** maturity-onset-diabetes of the young, MODY, pregnancy, monogenic diabetes, diabetes in pregnancy, GCK-MODY, HNF1A-MODY, HNF1B-MODY

## Abstract

Maturity-onset diabetes of the young (MODY)—a monogenic form of diabetes—accounts for approximately 1–2% of all diabetes cases, with GCK-MODY being the second most commonly diagnosed type. Although the inherited nature of the disease implies that the interplay between maternal glycemia and fetal genotype directly influences neonatal outcomes, clinical guidelines for MODY-complicated pregnancies remain underdeveloped. A systematic literature search in the PubMed, Scopus, Web of Science, and Cochrane databases was conducted following the PRISMA guidelines. The study protocol has been logged in the PROSPERO registry with the identification number CRD42024609390. Data, such as MODY type, the gestational age at delivery, mode of delivery, insulin administration, mutational status of the fetus, fetal birthweight (FBW), occurrence of small-/large-for-gestational age fetus, shoulder dystocia, and neonatal hypoglycemia, were extracted and evaluated. Among 19 studies selected for the final analysis, 15 investigated perinatal outcomes in the GCK-MODY variant. Women diagnosed with GCK-MODY treated with insulin delivered approximately 1–2 weeks earlier than those managed with diet alone. FBW was significantly higher in GCK-negative as compared to GCK-positive offspring. Accordingly, fetal macrosomia was notably more common among unaffected neonates. In GCK-affected fetuses, insulin therapy was associated with a significantly lower FBW. Fetal genotype critically modifies perinatal outcomes in GCK-MODY pregnancies. In the absence of fetal genotyping, conservative management should be prioritized to mitigate the risks of fetal growth restriction and iatrogenic prematurity. As data regarding other types of MODY in pregnancy remain sparse, there is an urgent need for more research in this area.

## 1. Introduction

Maturity-onset diabetes of the young (MODY)—a monogenic form of diabetes—accounts for approximately 1–2% of all diabetes cases, often being misdiagnosed as type 1, type 2, or gestational diabetes mellitus (GDM) [1]. To date, researchers have identified 14 distinct MODY subtypes, each associated with specific gene mutations that influence the function of pancreatic β-cells [2,3]. The most commonly diagnosed variant—MODY3—caused by a mutation in the hepatocyte nuclear factor 1-alpha (HNF1A) gene, accounts for roughly 69% of MODY cases, while the other two predominant types, glucokinase (GCK) and HNF4A, contribute to approximately 20% and 3%, respectively [2,4,5,6].

The HNF family comprises a group of transcription factors that play numerous roles in tissue development, as well as cellular metabolism regulation [7]. On the molecular level, both HNF1A and HNF4A interplay, as HNF4A positively regulates HNF1A expression, and HNF1A controls HNF4A binding to the P2 promoter in pancreatic cells [8]. Findings indicate that mutations affecting the HNF1A gene, located on 12q24, and the HNF4A gene, positioned at 20q12-13.1, lead to the reduced expression of the GLUT2 isoform of the facilitative glucose transporters, which ultimately results in severe hyperglycemia due to insufficient insulin secretion [8]. As a consequence, both proteins are essential for proper β-cell function and maintaining glucose homeostasis [9,10,11,12].

The second-most prevalent form of MODY-monogenic diabetes is associated with GCK, an enzyme that plays a pivotal role in the initial phase of glycolysis, specifically in converting glucose into glucose-6-phosphate through phosphorylation. Patients with GCK-MODY typically have much lower and more stable levels of hyperglycemia, usually ranging from 101 to 144 mg/dL, which is likely due to glucose regulation at a higher set-point [3,13]. Available data regarding other less common types of MODY in pregnancy are scarce. Nonetheless, it was established that HNF1B mutation often coexists with renal disorders or abnormal liver function, and patients typically need insulin as their therapeutic approach [14]. Similarly, PDX1-MODY, which is mostly diagnosed neonatally or in early adulthood, requires insulin administration during pregnancy to improve perinatal outcomes [14]. A summary of the most common variants of MODY-monogenic diabetes, including their genetic basis, prevalence, age of onset, treatment strategies, and associated complications, is provided in Table S1, adapted from Majewska et al. [14].

According to the literature, the monogenic type of diabetes is associated with multiple obstetric and neonatal complications, including fetal macrosomia, the delivery of a large-for-gestational-age (LGA) or small-for-gestational-age (SGA) fetus, preterm birth, neonatal hypoglycemia, and an increased rate of Cesarean section (CS) [15,16,17]. The genetic nature of the disease indicates that certain complications are contingent upon the specific MODY variant, the inheritance from the fetus, and the therapeutic interventions applied [14]. Evidence from recent studies reveals that fetuses with a positive GCK status are at a higher risk of being SGA, in contrast to GCK-negative fetuses, which are more likely to be overgrown [14]. It is important to note that, in the former cases, unjustified administration of insulin could increase the likelihood of giving birth to an SGA infant [18]. Finally, one must remember that, in the course of pregnancy, MODY is often misdiagnosed as GDM, and it is established that approximately 0.4–1.0% of patients diagnosed with GDM have, in fact, a monogenic type of diabetes [19]. As a result, the disease remains a significant diagnostic and therapeutic challenge, and more research is needed to properly manage the pregnancy in order to avoid associated complications.

Currently, there are no systematically collected data on the pregnancy outcomes in women with various types of MODY. Therefore, the present study aims to review and assess the maternal and fetal outcomes in pregnancies complicated by MODY-monogenic diabetes.

## 2. Materials and Methods

### 2.1. Search Strategy and Study Selection

A systematic literature search was performed in the PubMed, Scopus, Web of Science, and Cochrane databases for articles published between 1 January 2000 and 1 March 2025. The review was conducted following the Preferred Reporting Items for Systematic Reviews and Meta-Analyses (PRISMA) guidelines, and the study protocol was logged in the International Prospective Register of Systematic Reviews (PROSPERO) registry (no. CRD42024609390). The search strategy consisted of combinations of free text and MeSH terms, including “maturity-onset diabetes of the young”, “MODY”, “monogenic diabetes”, “glucokinase-MODY”, “GCK-MODY”, “hepatocyte nuclear factor, MODY”, “HNF, MODY”, “pregnancy”, “monogenic diabetes in pregnancy”, and “perinatal”.

Following the primary search, reference lists of selected studies were manually screened for other eligible publications. The inclusion criteria were randomized controlled trials (RCTs), observational studies, and case reports written in English. Given the low prevalence of disease, case reports and case series were considered significant, and evidence from such studies was included in the analysis. The exclusion criteria were studies regarding different types of diabetes, animal studies, and studies written in languages other than English. Importantly, since “monogenic diabetes in pregnancy” was used as a search term, studies on monogenic diabetes types other than MODY were not considered relevant for the data synthesis. The risk of bias in the selected studies was assessed independently by four researchers (F.Ł., J.B., K.M., P.J.S.) using the Downs and Black Checklist [20]. Any disagreements were resolved by the senior author (P.J.S.). Data verification was performed by P.J.S. and A.L. to ensure the accuracy and reliability of the extracted information.

Key domains included in the checklist, such as study quality (clarity and transparency of study objectives, outcomes, and findings), external validity (generalizability of the findings), internal validity (bias measurement and reliability of the reported outcomes, control of confounding variables, and appropriateness of statistical adjustments), and power analysis, were evaluated. RCTs would only be included if they achieved at least 24 out of 27 points, whereas for non-randomized studies, the score must have reached at least 11 out of 13 points. Following the initial screening, the preselected studies were further analyzed to assess final eligibility for the systematic review. No meta-analysis was performed due to significant disparity in the study populations and substantial heterogeneity in terms of study design, population size, and data reporting.

### 2.2. Data Extraction and Analysis

Titles and abstracts were screened independently by three researchers (F.Ł., J.B., K.M). The following information was collected: author’s name, year of publication, type of study, MODY variant, number of patients included in the analysis, number of offspring in total, number of MODY-affected offspring, number of MODY-unaffected offspring, number of MODY-affected offspring born to women treated with insulin, number of MODY-unaffected offspring born to women not treated with insulin, gestational age (GA) at delivery, fetal birthweight (FBW), delivery of a SGA/LGA infant, mode of delivery (CS/vaginal delivery—VD), insulin administration, occurrence of neonatal hypoglycemia (NH), shoulder dystocia (SD), preterm premature rupture of membranes (PPROM), and other perinatal outcomes.

### 2.3. Outcomes

The primary outcomes were FBW and GA at delivery. Numerous secondary outcomes were also analyzed, including the mode of delivery (CS/VD), administration of insulin therapy, delivery of an SGA/LGA infant, and the occurrence of SD and NH.

## 3. Results

**Figure 1:**
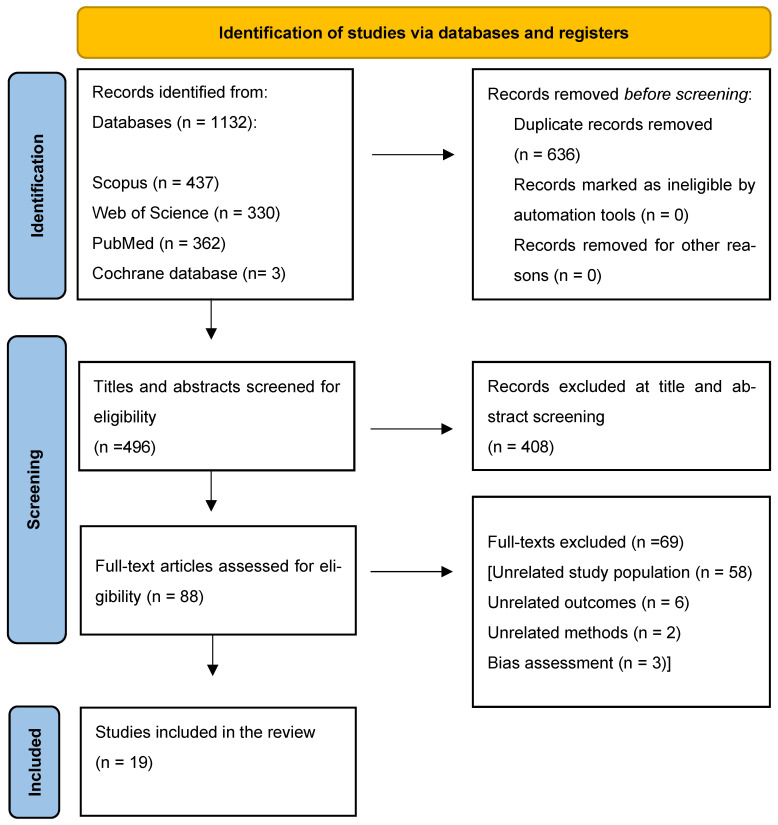
PRISMA flow chart of the screening process.

A total of 1132 articles were identified through a systematic review of the literature (Figure 1). After initial screening, 636 duplicates were excluded, and 496 titles and abstracts were further screened to evaluate eligibility. A total of 88 publications underwent an in-depth full-text analysis, resulting in 69 studies being excluded from further assessment. Among those 69 excluded studies, 3 were disqualified based on bias assessment results. Eventually, a total of 19 publications were included in this systematic review (Table 1).

### 3.1. Gestational Age at Delivery

Fifteen studies evaluated GA at delivery as an outcome, including eight case reports (Table 1) [15,17,21,22,23,24,26,29,30,31,32,33,34,35,36]. Of the studies cited, three analyzed GA contingent upon the treatment strategy for GCK-MODY [15,22,24], while four conducted a distinct analysis based on the offspring’s mutation status [16,24,25,27]. With regard to the treatment modality, Spyer et al. and De Las Heras et al. noted a lower GA at delivery in GCK(+) women receiving insulin during pregnancy compared to those solely on a diet (37.5 ± 1.7 weeks vs. 38.9 ± 2.3 weeks, *p* < 0.001; 37.8 ± 3.3 weeks vs. 39.6 ± 1.6 weeks, *p* = 0.05, respectively) [15,22]. Concerning merely fetal mutational status, in two studies, the authors did not report significant differences in GA between GCK-affected and unaffected offspring [15,23]. The remaining studies, which further classified the subjects into subgroups based on the presence of a mutation and treatment modality, failed to provide unambiguous results [23,24,26]. Firstly, Hosokawa et al. documented no significant differences in GA between GCK(+) and GCK(-) infants regardless of the mode of therapy [23]. On the contrary, Dickens et al. observed a significantly lower GA among GCK-affected offspring of insulin-treated mothers (38.0 vs. 40.4 weeks, *p* = 0.003), while no such differences in GCK-negative neonates were present (diet: 36 weeks vs. insulin: 37 weeks, *p* = 0.459) [24]. Finally, opposite results to the above mentioned, indicating a lower GA among insulin-treated GCK-negative fetuses, were reported by López Tinoco et al. (GCK-affected offspring—diet: 38.7 ± 1.4 vs. insulin: 39.6 ± 1.0 weeks, *p* = 0.07; GCK-unaffected offspring—diet: 39.5 ± 1.5 weeks vs. insulin: 38.3 ± 1.0, *p* = 0.03) [26].

Importantly, in a recent prospective study by Ciangura et al., the authors divided participants diagnosed with GCK-MODY into two groups in which the initiation of insulin therapy was decided based on measurements of (1) maternal capillary blood glucose (MG) or (2) fetal abdominal circumference by ultrasound (FG) [21]. No separate analysis for insulin-treated and non-insulin-treated patients was conducted. In the first group, the median GA was 38.9 [38.1–39.1], whereas in the second one, it was 38.7 [38.2–39.1] (*p* = 0.93) [21].

In the only observational study evaluating other than GCK-MODY variants of monogenic diabetes, the median GA at delivery in women diagnosed with HNF1A-MODY was significantly lower compared to GCK(+) individuals—39 weeks [38–40] vs. 40 weeks [39–40], respectively, (*p* = 0.02) [17].

Following the analysis of case reports, the mean GA at delivery for women with GCK-MODY was 37.8 ± 2.8 weeks [29,30,31]. Out of 16 deliveries, two (12.5%) occurred before 37 gestational weeks. Regarding types of monogenic diabetes other than GCK-MODY, Bitterman et al. and Mikuscheva et al. described cases of mothers with HNF1A-MODY, in whom labor occurred in the 37th and 35th week of gestation, respectively, [32,33]. Moreover, maternal HNF1B-MODY was described in three case reports [34,35,36]. There were six deliveries in total, including two premature, and the mean GA was 37.7 ± 1.5 weeks [34,35,36].

### 3.2. Fetal Birthweight

FBW as an outcome was evaluated in 10 retrospective [15,16,17,22,23,24,25,26,27,28] and 1 prospective study (Table 1) [21]. Out of those, three studies included a comparison of FBW depending on the mode of GCK-MODY treatment [17,22,24,25,26,28], whereas in seven, a separate analysis based on the offspring mutational status was performed [15,17,21,22,23,24,25,26,28]. In two studies by Kopacz-Petranyuk et al. and Bitterman et al., the authors did not distinguish between treatment methods or the MODY status of offspring [16,27].

According to the studies conducted by Spyer et al. and Bacon et al., the diabetes treatment modality did not influence the FBW in mothers with the GCK(+) genotype [17,22]. The latter study reported a mean FBW of 3900 g (3200–4500) among insulin-treated women vs. 3600 g (2800–3600) in those solely on a diet (*p* = 0.6), whereas the former documented FBWs of 3700 ± 700 g vs. 3800 ± 600 g, respectively, (*p* = 0.1). Conversely, in the study by Fu et al., the administration of insulin in GCK-affected women was associated with a significantly lower FBW (2830 g vs. 3370 g, *p* = 0.003) [25].

The comparative analysis between GCK(+) and GCK(-) neonates unambiguously revealed that unaffected offspring were significantly heavier than mutation carriers [15,17,22,23,26,28]. It is important to note that, according to Dickens et al., children of women treated with insulin during pregnancy and carrying a GCK mutation demonstrated a notable decrease in their FBW (2967 ± 933 g vs. 3725 ± 568 g, *p* = 0.005) [24]. The same has not been observed among GCK-unaffected infants, where the mean FBW was 3757 ± 532 g and 4023 ± 284 g, respectively, (*p* = 0.489). Similar observations regarding the impact of insulin therapy on the FBW in GCK(+) offspring were reported by two other authors [22,28].

Results from the prospective study by Ciangura et al. demonstrated that the mode of pregnancy management did not affect FBW among GCK(+) women [21]. In the subgroup where only maternal glycemia was monitored, the median FBW reached 3340 g [3040–3660], whereas in patients with additional ultrasound surveillance, it was 3265 g [3125–3640] (*p* = 0.95).

Bacon et al., who compared populations with GCK-MODY and HNF1A-MODY variants, observed a mean FBW of 3900 g (3200–4500) and 3600 g (3100–4000), respectively, (*p* = 0.4) [17]. Notably, with regard to the latter cohort, the mode of therapy did not alter FBW—insulin, 3200 g (3200–3900) vs. diet, 3600 g (3200–4000) (*p* = 0.5).

In case reports, the mean FBW for term singleton offspring born to women with GCK-MODY and HNF1B-MODY was 3126.0 ± 565 g and 3479.8 ± 655 g, respectively, [29,30,31]. There were no reports on singleton term neonates in women with the MODY3 variant [32,33].

### 3.3. Fetal Macrosomia or Large-for-Gestational-Age Infant

Six original studies reported fetal macrosomia in GCK(+) mothers [15,17,22,23,27,28], whereas four evaluated LGA (Table 1) [16,21,24,26]. Fetal macrosomia was observed in 49 neonates, which corresponds to 15.3% of all infants born to GCK(+) mothers, and 49 cases (29.9%) were classified as LGA. Additionally, Dickens et al. reported 11 (31.4%) cases of LGA among infants with a confirmed mutation status [24]. An independent assessment of macrosomia/LGA prevalence in the GCK(+) and GCK(-) offspring was undertaken in seven distinct studies [15,17,22,23,24,26,28]. In all publications, the authors reported a higher prevalence of fetal macrosomia/LGA among GCK-unaffected children [15,17,22,23,24,26,28]. Regarding fetal macrosomia, the detected rates fluctuated between 16% and 40.9% in infants without the GCK mutation, compared to a mere 0% to 8.9% in those affected [15,22,23,28]. Consistent with these findings, in the studies by López-Tinoco et al. and Dickens et al., LGA was observed in 65.2% and 50% of GCK(-) infants, and in 12.8% and 21.7% of mutational carriers, respectively, [26]. Finally, the study conducted by Kopacz et al. revealed a significant decrease in macrosomia rates among children with GCK-MODY, compared to the general pediatric population (*p* = 0.02) [27].

With regard to treatment modality, Dickens et al. observed LGA in 56% of insulin-treated and in 33% of untreated GCK-negative infants (*p* = 0.59), whereas among GCK(+) offspring, LGA occurred only in five (33.3%) neonates whose mothers had not received insulin [24]. Similar pattern of fetal growth in GCK(-) offspring was reported by López Tinoco et al.: LGA occurred in 6 (50%) of neonates born to mothers who were not given insulin and in 9 (82%) neonates of insulin-treated mothers (*p* = 0.12) [26]. Among GCK-affected offspring, LGA occurred in one (4%) and four (36%) cases, respectively, (*p* = 0.06). Lastly, regarding fetal macrosomia, in the study by Bacon et al., the authors observed no cases of fetal macrosomia among the GCK-affected offspring regardless of the mode of treatment [17]. However, in the unaffected cohort, the prevalence was higher and reached 62.5% in children born to diet-controlled mothers and 33.3% in the insulin-treated group, (*p* = 0.8).

Remarkably, in the publication by Ciangura et al., the authors analyzed two separate approaches to managing GCK-MODY (i.e., the initiation of insulin therapy based on maternal blood capillary glucose measurements, MG vs. ultrasound assessment of fetal growth, FG) and concluded that there was no significant disparity in LGA rates between the MG and FG groups—6 (24.0%) vs. 4 (20.0%), respectively, [21].

The only observational study assessing the incidence of fetal macrosomia in women with HNF1A-MODY reported that it was significantly less frequent in HNF1A, as compared to GCK-MODY (10% vs. 31.2%, *p* = 0.01) [17]. Moreover, in the former group, a higher rate of macrosomia (25%) was observed among the diet-controlled HNF1A(+) offspring compared to those managed with pharmacological treatment.

The findings from the non-observational studies included three cases of fetal macrosomia and/or LGA [34]. Morton et al. documented LGA in three infants born to mothers with the MODY-5 variant [34]. One of these infants had a FBW exceeding 4000 grams, thus meeting the criteria for macrosomia.

### 3.4. Small-for-Gestational-Age Infant

A total of five observational studies assessed the occurrence of SGA as an outcome, revealing that it was present in seven cases (3.74% of the total number of neonates) (Table 1) [16,17,21,23,28]. Additionally, López Tinoco et al., who evaluated exclusively neonates with a confirmed mutational status, observed SGA in five (8.1%) cases [26].

The separate risks of SGA in GCK(+) and GCK(-) offspring were reported in three studies [17,23,26]. In the affected neonates, SGA occurred in six (7.5%) cases, whereas among non-carriers, SGA was present in a single infant (2.2%) [17,23,26]. None of the studies found statistically significant differences between the groups with regard to the incidence of SGA. Concerning the mode of treatment and SGA occurrence, Hosokawa et al. documented a single case of an SGA GCK-affected neonate born to an insulin-treated mother, whereas López Tinoco et al. reported four (14%) and zero (0%) cases of an SGA GCK(+) infant in diet- and insulin-controlled women, respectively, (*p* = 0.3) [23,26]. Lastly, in the prospective study by Ciangura et al., SGA occurred in one (5.0%) infant in the FG group, with no cases in the MG group [21].

The analysis of FBW percentiles documented in case reports revealed the presence of SGA in three infants [29,31,34]. Two of them occurred in GCK-MODY [29,31] and one in HNF1B-MODY variants of monogenic diabetes [34].

### 3.5. Neonatal Hypoglycemia

In four separate studies, researchers examined NH as an outcome in the entire study population, leading to the identification of 16 cases, accounting for 6.8% of the overall findings (Table 1) [17,21,26,27]. Only in two studies was the outcome assessed based on the MODY status of the neonate [24,26]. Dickens et al. documented a single case of NH in a GCK(+) fetus born to an insulin-treated mother, with no analogous cases present in other groups [24]. In the study by López Tinoco et al., the authors found four (36%) and two (17%) cases of NH in GCK-unaffected offspring born to insulin- and diet-treated women, respectively, (*p* = 0.37) [26]. Among affected children the observed rates were lower, however, this was without significant differences between both treatment modes—two (18%) and zero (0%) cases, respectively, (*p* = 0.07) [26]. Finally, in the single prospective study by Ciangura et al., the authors documented two cases(9%) and one (5%) case of NH in the MG and FG groups, respectively, [21].

Regarding women with the HNF1A-MODY variant, a non-significant increase in the incidence of NH amongst unaffected offspring not treated with insulin was noted [17].

NH was reported in four infants in case studies [30,32,34]. Udler et al. identified two cases of GCK-MODY-associated NH [30], whereas the other two occurred in neonates born to mothers with HNF1A- and HNF1B-MODY variants [32].

### 3.6. Shoulder Dystocia

Two observational studies analyzed SD as an outcome in the full study cohort, revealing its presence in seven (5.5%) cases (Table 1) [21,22]. Two other studies assessed SD within the subgroup of infants with a confirmed mutation status and reported four cases, which corresponds to 4.7% of the total [17,26]. López Tinoco et al. reported three (3.0%) cases of this adverse perinatal outcome, while Bacon et al. observed only a single case of SD in their population (4.3%). In addition, Spyer et al. observed SD in four (26.7%) macrosomic, GCK-unaffected offspring, which translates into 4.9% of all offspring born to GCK(+) mothers [24].

In the remaining study by Ciangura et al., the authors documented two (9%) cases of SD in the group of GCK-MODY (+) women in whom insulin was administered based on capillary blood glucose measurements and one (5%) case among women in whom insulin was introduced following the assessment of fetal growth [21].

No case reports included a description of SD.

### 3.7. Mode of Delivery

Six original studies reported the rate of CS (Table 1) [15,17,21,22,24,26]. In the studies by Spyer et al. and Dickens et al., the authors observed a total of 11 (26%) and 5 (14.3%) CSs among GCK-affected women [22,24]. Analysis of separate populations of GCK(+) and GCK(-) infants by Lopez Tinoco et al. revealed that, in the non-affected offspring, surgical delivery was performed significantly more often compared to mutation-carriers—17 (74%) vs. 11 (28%), respectively, *p* = 0.001 [26]. In addition, among GCK(-) fetuses, the rate of CS was higher in those receiving pharmacological treatment compared to those managed solely with diet; however, the difference was not statistically significant (82% vs. 67%, *p* = 0.64). Contrarily to the previous study, De las Heras et al. observed no significant difference in the rate of CS between affected and non-affected offspring of GCK(+) mothers—11 (24.4%) vs. 5 (22.7%), respectively [15]. Lastly, in the study designed to compare two management strategies for GCK-MODY, researchers noted that the CS rates for the MG and FG groups were similar—11 (44%) vs. 7 (35%), respectively, (*p* = 0.65) [21].

In the only study comparing GCK-MODY and HNF1A-MODY cohorts, Bacon et al. observed a higher incidence of CS in the former [17]. Among women treated with insulin, CS was performed in 57.2% and 37.5%, respectively. Likewise, in individuals not undergoing pharmacological therapy, the incidence of surgical deliveries was higher in patients with GCK-MODY—42.8% vs. 14.3%. Both observed differences, however, were not statistically significant [17].

In case studies, CS was reported in three publications, and a total of five surgical procedures were performed [29,32,34]. There were three emergency surgical deliveries [29,34]. Six neonates were delivered via CS, including two infants born prematurely (one with GCK-MODY, two with HNF1A-MODY, three with HNF1B-MODY). Regarding VD, five publications reported a total of six labors, including three spontaneous deliveries [31,33,34,35,36]. Half of VDs occurred before 37 gestational weeks [31,33,35].

### 3.8. Insulin Therapy

Nine original studies documented data on insulin therapy in pregnancies with concomitant monogenic diabetes (Table 1) [15,17,21,22,23,24,26,28]. Among these, three studies reported data on the average time of pharmacological treatment initiation [17,24,26]. According to the analysis, 48.3% of mothers diagnosed with GCK-MODY received insulin at an average GA of 17.0 ± 3.2 weeks [17,24,26]. Separate cohorts of GCK(+) and GCK(-) offspring were analyzed by López Tinoco et al. and Bacon et al. [17,26]. Both studies demonstrated higher rates of insulin administration among GCK-negative infants, 47.8% and 30.0%, compared to GCK-affected offspring, 28.2% and 23.1%, respectively [17,26].

The single study by Bacon et al. evaluated diabetes treatment among GCK- and HNF1A-MODY-affected women [17]. The percentage of patients receiving insulin during the course of pregnancy was comparable between both MODY variants—26.0% in HNF1A-MODY, compared to 26.6% of GCK-affected individuals. The average GA of pharmacological treatment initiation in HNF1A(+) women was higher than in GCK(+)—15 weeks vs. 14 weeks, respectively. The difference, however, was not significant.

Ciangura et al. examined the timing of treatment initiation according to the specific trimester during pregnancy and the method of patients’ surveillance [21]. In that study, insulin therapy was commenced prior to pregnancy in 3 (7.5%) women, 23 (57.5%) started receiving insulin in the first trimester, 8 (20.0%) in the second, and 6 (15.0%) in the third. It is of particular relevance to note that insulin therapy was initiated both more frequently (*p* = 0.01) and at an earlier GA (*p* = 0.001) in the group of women who exclusively monitored blood capillary glycemia, compared to the FG group. In the MG cohort, all participants required pharmacological treatment: 3 commenced therapy prior to conception, 18 during the first trimester, and 4 in the second trimester. In contrast, in the FG group, insulin was administered to 15 women (75%). Among them, seven initiated treatment due to a fetal abdominal circumference ≥75th percentile on ultrasound (median GA 29.7 weeks [25.7–35.3]), seven due to elevated capillary blood glucose levels (median GA 13.1 weeks [12.0–22.6]), and one woman commenced insulin for both indications at 33 + 0 weeks of gestation. The study findings indicated also that the initiation time for insulin therapy was similar for both LGA and non-LGA infants (*p* = 0.71), as pharmacological treatment was initiated in 60% and 57% of mothers in the first trimester, respectively. During the second trimester, insulin therapy was commenced in 30% and 17% of cases, while in the third trimester, initiation rates were 10% and 17%, respectively.

Insulin was administered to all mothers in case studies [29,30,31,32,33,34,35,36]. The time of insulin therapy initiation was reported in five cases, with an average GA of 9.4 ± 2.8 weeks and 12 weeks among women with GCK-MODY and HNF1B-MODY, respectively, [29,30,31,34].

## 4. Discussion

The systematic review of the literature led to the identification of 10 retrospective cohort studies, 1 prospective cohort study, and 8 case studies that explore perinatal outcomes across different maternal MODY variants. The evaluation of the results suggests that the approach to care for pregnant women is contingent upon the specific MODY classification and the existence of a mutation in the fetus [14]. The latter is most evident among women with the GCK-MODY variant, as it has a significant impact on both the FBW and the GA at delivery. The explanation for the above phenomenon stems from pathophysiological mechanisms that are specific to fetuses of GCK-MODY-affected mothers. Evidence shows that in fetuses carrying a *GCK* mutation, the stimulation threshold of glucose-dependent β-cells’ insulin secretion is increased [37]. As a consequence, the release of insulin and fetal growth remain relatively unaltered in the hyperglycemic environment [37]. On the contrary, in fetuses without a *GCK* mutation, β-cells respond to maternal hyperglycemia with greater insulin production, which ultimately leads to the fetus’s overgrowth [37]. This genotype–phenotype interaction underscores the importance of fetal genotyping in guiding therapy, which has been advocated in the previous literature but remains underutilized in routine clinical practice due to logistical constraints and cost concerns [37,38].

Our results highlight the increased risk of fetal macrosomia/LGA in GCK-negative offspring, particularly when maternal glycemia is suboptimally controlled [15,17,22,23,24,26,28]. This finding aligns with the above-mentioned pathophysiological model of increased fetal insulin production in response to maternal hyperglycemia and reinforces the need for precise glucose monitoring in these pregnancies. In contrast, a higher rate of SGA was noted among GCK-positive (7.5%) compared to GCK-negative offspring (2.2%), although this difference was not statistically significant in any of the studies that stratified the population by fetal genotype [17,23,26]. Nonetheless, these observations highlight an association between the presence of a GCK mutation and constrained fetal growth, particularly in the context of insulin therapy.

Our findings confirm previous hypotheses that insulin therapy may not be beneficial, and in fact could be detrimental, in pregnancies where the fetus inherits the GCK mutation. Several studies included in this review demonstrated a lower FBW in GCK(+) offspring of insulin-treated mothers [22,24,25]. In addition, available data imply that doses of insulin required for proper glycemic control may be higher in GCK(+) fetuses, which could result in an increased frequency of hypoglycemic events [38]. These observations suggest potential overtreatment and raise critical issues surrounding the indiscriminate prescription of insulin without the benefit of fetal genotyping. On the other hand, diet-controlled pregnancies consistently achieved term deliveries with favorable neonatal outcomes, further supporting conservative management in genetically confirmed cases.

Another important observation is the variation in GA at delivery associated with insulin administration. In several studies, insulin-treated mothers diagnosed with MODY-monogenic diabetes delivered earlier than their diet-managed counterparts [22,23,24,26]. While this may be attributed to closer monitoring and a lower threshold for intervention in women requiring insulin, it raises concerns about potential iatrogenic prematurity, particularly when fetal growth is not excessive. In relation to the most extensively analyzed variant of monogenic diabetes, GCK-MODY, available data suggest that GA is associated with both the type of treatment and fetal mutational status [22,23,24,26]. Although in general, better pregnancy outcomes are achieved with diet control and in fetuses without the causative mutation, current findings suggest that in insulin-treated mothers, term labor could be achieved in the majority of cases [15,21,22,23,24,26].

Regarding the mode of delivery, available data indicate that the CS rate is often higher among women in whom insulin therapy was initiated [15,17,26]. The question of whether this observation is linked to obstetric indications (e.g., macrosomia, shoulder dystocia risk) or influenced by clinical biases in handling high-risk pregnancies remains unresolved. One can confidently assert that the delivery approach should be personalized based on the parameters of fetal growth, the patients’ obstetric history, and the current pregnancy course, rather than focusing exclusively on the maternal MODY status.

NH appears to be an infrequent complication in pregnancies affected by MODY-monogenic diabetes [21,24,26,27]. Despite limited data, available evidence suggests that NH is more common in GCK-unaffected neonates, particularly when insulin therapy is administered [24,26]. The observed pattern provides additional evidence that maternal hyperglycemia enhances fetal insulin synthesis among GCK(-) offspring, leading to neonatal complications associated with fetal overgrowth.

The prospective study by Ciangura et al. is the only available investigation to directly compare two management strategies for GCK-MODY pregnancies: the initiation of insulin therapy based on measurements of maternal capillary blood glycemia vs. fetal ultrasound surveillance [21]. Although they were non-significant, obtained results demonstrated that ultrasound-guided management, initiated in response to accelerated intrauterine fetal growth, was associated with an earlier GA at delivery and a lower FBW. Notably, the ultrasound-monitored group had fewer cases of LGA, SD, NH, and CS, suggesting a possible reduction in obstetric and neonatal complications. These findings emphasize the potential benefits of individualized, growth-based monitoring over purely glycemia-driven treatment in GCK-MODY pregnancies.

Conversely to GCK-MODY, in the most frequently occurring HNF1A-MODY (MODY3) variant, pregnancy outcomes are largely dictated by the glycemic regulation of the mother instead of the fetal mutation status [14]. This underscores the importance of achieving euglycemia early in pregnancy, as maternal hyperglycemia in the first trimester has been associated with an increased risk of congenital malformations and, in more advanced stages of gestation, directly influences fetal growth [14,39]. Importantly, in the only study involving a larger cohort of individuals with HNF1A mutations, no significant differences in FBW were observed between insulin- and non-insulin-treated groups, and fetal macrosomia occurred considerably less frequently compared to GCK-MODY [17]. Very little obstetric data is available regarding other types of monogenic diabetes. Similarly to MODY3, the scarce evidence suggests a requirement for insulin therapy for glycemic control in HNF4A (MODY1), PDX1 (MODY4), and HNF1B (MODY5) variants, rather than an attempt at diet control [14].

To summarize, the presented study offers a synthesis of current knowledge on pregnancy outcomes in women affected by MODY, a rare and often misdiagnosed form of monogenic diabetes. One of the key strengths of this systematic review is its transparent methodology, including its adherence to PRISMA guidelines and a registered PROSPERO protocol. In addition, the inclusion of studies addressing various MODY subtypes adds breadth and relevance, particularly given the limited existing literature on the topic. Finally, compared to previous reviews, the current work incorporates the most recent studies, thus reflecting the up-to-date state of evidence. Nonetheless, certain limitations need to be addressed. First, not all MODY subtypes were represented in the analysis, largely due to their extremely low prevalence and the lack of related publications in the obstetrics population. Furthermore, only one original study addressing HNF1A-MODY was identified, highlighting a gap in the literature for this variant. The absence of RCTs is another significant limitation; as no such studies were identified through the literature search, the conclusions drawn are based solely on observational data and case reports, which may introduce bias and limit the generalizability of the findings.

## 5. Conclusions

The results of this systematic review support prior research indicating that the fetal genotype plays a pivotal role in determining perinatal outcomes in pregnancies complicated by MODY-monogenic diabetes, in particular among GCK-MODY-affected women. While those observations advocate for genotype-guided management, the scarcity of fetal genotyping in conventional clinical practice limits the ability to accurately diagnose and manage atypical cases of maternal hyperglycemia during pregnancy. Finally, as data on non-GCK-MODY subtypes, in particular HNF1A and HNF1B, remain sparse, further research is needed to delineate their pregnancy-related risks. Until more robust evidence emerges, treatment strategies should balance glycemic control with careful monitoring of fetal growth, prioritizing non-invasive approaches where possible.

## Figures and Tables

**Table 1:** Characteristics of studies included in the analysis.

Author, Year and Country	Study Type	MODY Variant	Number of Patients	Number of Offspring	Number of MODY(+) Offspring	Number of MODY(+) Offspring Born to Women Treated with Insulin	Number of MODY(-) Offspring	Number of MODY(-) Offspring Born to Women Not Treated with Insulin	Analyzed Outcomes	Results
Ciangura et al., 2025, France [21]	Prospective observational	GCK-MODY	45	45	18	15	25 and 2 with an undetermined status	2	FBW, GA, LGA, SGA, NH, SD, mode of delivery	The mean GA at delivery was higher in the MG group, compared to the FG cohort—38.9 weeks (range: 38.1–39.1) vs. 38.7 weeks (range: 38.2–39.1), *p* = 0.93. The median FBW was higher in the MG group—3340 g [3040–3660] vs. 3265 g [3125–3640], *p* = 0.95. In the MG cohort, there were no SGA fetuses, compared to 1 (5%) in the FG group, *p* = 0.44. In the MG cohort, LGA occurred in 6 (24%), whereas in the FG, it occurred in 4 (20%) neonates, *p* > 0.99. SD was more frequent in the MG group—2 (9%) vs. 1 (5%). In the MG group, NH requiring treatment was reported in 2 (9%), whereas in the FG, it was reported in 1 (5%) neonate. In the MG group, CS was performed in 11 (44%), and in the FG, it was performed in 7 (35%) cases, *p* = 0.65.
Spyer et al., 2009, United Kingdom [22]	Retrospective observational	GCK-MODY	42	82	44	14	38	19	FBW, GA, fetal macrosomia, SD, mode of delivery, insulin administration	GCK-unaffected offspring had a higher mean FBW than mutation-carrying offspring (3900 ± 600 g vs. 3200 ± 800 g, *p* < 0.001) and was more likely to be macrosomic (39% vs. 7%, *p* = 0.001). The mean FBW of GCK-MODY-affected children was 3000 ± 900 g following insulin treatment, and 3300 ± 700 g on diet, *p* < 0.05. Among GCK-unaffected children, the mean FBW in the insulin-treated cohort was 3800 ± 600 g, and 4000 ± 500 g in the diet-controlled group, *p* < 0.001. No difference in the FBW between insulin- and diet-treated women was reported (3700 ± 700 g vs. 3800 ± 600 g, *p* = 0.1), respectively. The mean GA was lower in insulin-treated mothers when compared to those on a diet only (37.5 ± 1.7 weeks vs. 38.9 ± 2.3 weeks, *p* < 0.001), respectively. Four (26.7%) of the macrosomic GCK-unaffected infants presented with SD. CS rate was higher in GCK(+) women compared to those without the mutation, 26% vs. 3%, respectively.
Hosokawa et al., 2019, Japan [23]	Retrospective observational	GCK-MODY	23	40	28	9	12	3	FBW, GA, fetal macrosomia, SGA, insulin administration	GA at delivery was lower in the GCK-affected as compared to unaffected offspring 38.4 ± 2.3 weeks vs. 39.3 ± 1.0 weeks, respectively, *p* = 0.45. GA was lower in the GCK-affected offspring in the insulin-treated group compared with the diet-controlled cohort—37.6 weeks vs. 38.8 weeks, respectively, *p* = 0.308. For unaffected offspring, the mean GA was 39.4 weeks vs. 39.3 weeks, respectively, *p* = 0.933. Significantly higher FBW in the unaffected as compared to GCK-affected offspring was reported—3403 ± 559 g vs. 2713 ± 551 g, *p* = 0.005. Fetal macrosomia occurred in 0 (0%) of GCK-affected and 2 (16%) of unaffected offspring, *p* = 0.002. A single GCK-affected neonate born to an insulin-treated woman was SGA.
Bacon et al., 2015, Ireland [17]	Retrospective observational	GCK-MODY, HNF1A-MODY	37 (12 GCK-MODY, 25 HNF1A-MODY)	132 (106 live births—41 in the GCK-MODY group and 65 in the HNF1A-MODY group) * * Genetic testing performed in 23 neonates of GCK-affected mothers.	13 with GCK-MODY, no data available for HNF1A-MODY	3 with GCK-MODY, no data available for HNF1A-MODY	10 with GCK-MODY, no data available for HNF1A-MODY	7 with GCK-MODY, no data available for HNF1A-MODY	FBW, GA, fetal macrosomia, NH, mode of delivery, insulin administration, SD, SGA	Median FBW in the unaffected offspring was significantly higher compared to the GCK-affected (4800 g [4100–5200] vs. 3200 g [3100–3700], *p* = 0.01), respectively. The comparison of populations with GCK-MODY and HNF1A-MODY variants revealed a mean FBW of 3900 g (3200–4500) and 3600 g (3100–4000), respectively, *p* = 0.4. No significant difference was found between the insulin treated and non-insulin treated groups regarding mean FBW in GCK-affected pregnancies when mutation status of the offspring was not considered 3900 g (3200–4500) vs. 3600 g (2800–3600), respectively, *p* = 0.6. In GCK-affected offspring, no cases of fetal macrosomia were noted regardless of the treatment method. In GCK-unaffected offspring a higher incidence of fetal macrosomia was observed in diet-controlled mothers compared to pharmacological treatment (62.5% vs. 33.3%, *p* = 0.8). Fetal macrosomia was significantly less frequent in the HNF1A cohort, as compared to GCK-MODY (10% vs. 31.2%, *p* = 0.01) In the HNF1A group, no difference between the insulin and non-insulin treated women with regard to mean FBW was noted 3200 g (3200–3900) vs. 3600 g (3200–4000), respectively, *p* = 0.5. There was a higher incidence of fetal macrosomia amongst the HNF1A-affected offspring not treated with insulin (25%). In addition, there was a higher incidence of prolonged hypoglycemia (16%) amongst HNF1A-unaffected offspring not treated with insulin—differences were not significant. Median GA at delivery was higher in the GCK-MODY cohort, as compared to HNF1A mothers—(40 [39–40] weeks vs. 39 [38–40] weeks, *p* = 0.02). Higher incidence of CS was noted in GCK-MODY cohort compared to HNF1A-MODY. Among insulin-treated women: 57.2% vs. 37.5%, whereas in diet-controlled women: 42.8% vs. 14.3%—the differences were not significant. Among GCK-unaffected offspring treated with diet, complications were observed in 3 out of 7 cases (37.5%). These included a congenital neural tube defect, SD, and extended episodes of NH. There was a single case of SGA in GCK(+) offspring, and none in GCK(-).
Dickens et al., 2019, United States [24]	Retrospective observational	GCK-MODY	54	128 ** ** Genetic testing performed in 37 neonates, out of whom, in 35 a mutation was confirmed.	23	8	12	4	FBW, GA, LGA, mode of delivery, insulin administration, NH	Mean FBW in GCK-affected infants was significantly lower with maternal insulin treatment compared to no treatment (2967 ± 933 g vs. 3725 ± 568 g, *p* = 0.005). Among GCK-unaffected infants the mean FBW was 3757 ± 532 g in the insulin-treated group, and 4023 ± 284 g in the diet-controlled cohort (*p* = 0.489). Overall, 27% of infants were classified as LGA. Among insulin-treated mothers, 38% of infants were LGA, compared to 23% among non-insulin-treated women, *p* = 0.04. LGA occured in 56% of GCK-unaffected infants treated with insulin, and in 33% on diet, *p* = 0.59. The mean FBWs in the aforementioned cohorts were 3757 ± 532 g and 4023 ± 284 g, respectively, *p* = 0.489. Among GCK(+) neonates, LGA occurred in 5 (33.3%) treated with diet. GA at delivery was significantly lower in the GCK-affected infants in the maternal insulin treatment group compared to no treatment—38.0 weeks vs. 40.4 weeks, respectively, *p* = 0.003. There was no significant difference in GA at delivery for GCK-unaffected infants based on treatment (37 weeks with insulin vs. 36 weeks without insulin treatment, *p* = 0.459). The average GA at delivery for the entire cohort was 38.8 weeks, with no significant difference observed between insulin-treated and non-insulin-treated groups. NH occurred in a single infant born to a GCK-MODY(+) mother treated with insulin. Among insulin-treated, GCK(-) neonates, 2 out of 9 (22%) required CS. Similarly, in the untreated GCK(+) group, 2 out of 15 (13%) neonates were delivered by CS.
Fu et al., 2019, China [25]	Retrospective observational	GCK-MODY	n/a	n/a	28	7	n/a	n/a	FBW, insulin administration	The mean FBW of neonates born to GCK-MODY women was 3110 ± 440 g. Neonates whose mothers were treated with insulin had significantly lower birthweight (2830 ± 390 vs. 3370 ± 390, *p* = 0.003).
López Tinoco et al., 2021, Spain [26]	Retrospective observational	GCK-MODY	34	119 (99 live births)	39	11	23	11	FBW, GA, LGA, SGA, mode of delivery, insulin administration, SD, NH	FBW, percentage of LGA and CS in GCK-unaffected offspring was significantly higher than in GCK-affected at 4000 ± 700 g vs. 3400 ± 400 g, *p* = 0.001; 15 (65%) vs. 5 (13%), *p* < 0.001, and 17 (74%) vs. 11 (28%), *p* = 0.001, respectively. An earlier GA at delivery was observed on insulin in GCK-unaffected offspring 38.3 ± 1.0 weeks vs. 39.5 ± 1.5 weeks, *p* = 0.03; with no significant change in the rate of CS—9 (82%) vs. 8 (67%), *p* = 0.64; and LGA—9 (82%) vs. 6 (50%), *p* = 0.12, compared to the diet-controlled cohort. No differences in the GA and rate of SGA in the GCK-affected offspring following insulin therapy were noted compared to diet only—(39.6 weeks vs. 38.7 weeks, *p* = 0.07), and 0 (0%) vs. 4 (14%), respectively, *p* = 0.3. SD was observed in 3 neonates. NH was observed in 2 cases (18%) among insulin-treated, GCK-affected offspring, and in none (0%) of the non–insulin-treated counterparts (*p* = 0.07). In the GCK-unaffected group, NH occurred in 4 neonates (36%) receiving insulin therapy and in 2 (17%) without pharmacologic treatment (*p* = 0.37).
Kopacz-Petranyuk et al., 2018, Poland [27]	Retrospective observational	GCK-MODY	n/a	50	32	n/a	n/a	n/a	FBW, fetal macrosomia, NH	The mean FBW was 3356.8 ± 557.53 g. Fetal macrosomia was significantly less frequent in children with GCK-MODY in comparison to the general pediatric population (*p* = 0.02). No episodes of NH were recorded.
Jiang et al., 2022, China [28]	Retrospective observational	GCK-MODY	41	41	32	6	9	5	FBW, fetal macrosomia, SGA, insulin administration	The mean FBW in the GCK-affected offspring was lower compared to unaffected 3195.5 g vs. 3734.4 g, *p* < 0.001. There was a significant difference in the FBW of GCK-affected infants born to insulin-treated mothers—3176 ± 166.4 g compared to the diet-controlled group—3295.7 ± 182 g, *p* = 0.03. No difference in FBW was found for GCK-unaffected infants (insulin: 3567.5 ± 693.5 g vs. diet: 3868.0 ± 399.2 g, *p* = 0.47). Fetal macrosomia was observed in 33.3% (3/9) of GCK-unaffected, and in none of the affected offspring, (*p* = 0.008). Two cases of SGA were reported in the offspring of affected mothers treated with insulin. No perinatal complications occurred in the diet-treated group.
De las Heras et al., 2010, Spain [15]	Retrospective observational	GCK-MODY	31	67	45	10	22	8	FBW, GA, fetal macrosomia, mode of delivery	Mean FBW in the GCK-affected offspring was lower compared to those unaffected, 3126 ± 666 g vs. 3760 ± 1103 g, respectively, *p* = 0.004. GA at delivery did not differ significantly between both groups. GCK-affected offspring were born at 39.3 ± 2.3 weeks and unaffected at 38.7 ± 2.7 weeks. GCK(+) mothers who received insulin therapy delivered an average of 1.8 weeks earlier compared to those managed with dietary treatment (37.8 ± 3.3 weeks vs. 39.6 ± 1.6 weeks, *p* = 0.05). Fetal macrosomia was significantly more common in the GCK-unaffected compared to affected offspring, 40.9% vs. 8.9%, respectively, *p* = 0.006. CS was performed in 22.7% of GCK-unaffected and 24.4% of affected offspring (not significant).
Bitterman et al., 2018, Italy [16]	Retrospective observational	GCK-MODY	n/a	20	20	0	n/a	n/a	FBW, SGA, LGA	Mean FBW was 3130 g (2910–3500). LGA occurred in 3 cases (15%), whereas SGA occurred in 2 (10%).
Yau et al., 2022, China [29]	Case report	GCK-MODY	1	1	n/a	n/a	n/a	n/a	FBW, GA, mode of delivery, SGA	Emergency CS at 33 weeks of gestation due to PPROM. FBW of 1815 g (<3rd percentile).
Udler et al., 2020, United States [30]	Case report	GCK-MODY	1	4	1	1	3	3	FBW, GA, NH. mode of delivery	Labors occurred between 38 and 39 + 3 weeks of gestation; children weighed between 2600 and 3900 g. NH occurred in 2 (50%) of infants.
Spyer et al., 2001, United Kingdom [31]	Case report	GCK-MODY	1	2	1	1	1	0	FBW, GA, mode of delivery, SGA	Induced VD at 36 weeks of gestation. FBW of 1610 g (0 percentile). Second labor induced at 37 weeks. FBW of 2630 g (30th percentile).
Bitterman et al., 2016, Italy [32]	Case report	HNF1A-MODY	1	2	1	1	1	0	FBW, GA, NH, mode of delivery	Elective CS at 37 weeks of gestation. Two neonates were born. FBW of 2600 g (no mutation) and 2660 g (mutation carrier). Transient NH occurred in the mutation-carrying newborn.
Mikuscheva et al., 2018, Czech Republic [33]	Case report	HNF1A-MODY	1	1	0	0	0	0	FBW, GA, mode of delivery	VD at 35 weeks of gestation. FBW of 2220 g.
Morton et al., 2022, United Kingdom [34]	Case report	HNF1B-MODY	3	4	1 (confirmed)	1	2	0	FBW, GA, fetal macrosomia, LGA, NH, mode of delivery, SGA	Case 1: Emergency CS at 37 weeks of gestation due to preeclampsia. FBW of 3400 g (97th percentile). Elective repeat CS at 37 weeks of gestation. FBW of 4413 g (100th percentile). Case 2: Induced VD at 37 weeks of gestation. FBW of 3206 g (90th percentile). Neonatal course was complicated by hyperinsulinemic hypoglycemia requiring intravenous glucose. Case 3: Emergency CS at 25 + 4 weeks of gestation due to placental abruption FBW of 602 g (5th percentile).
Mikuscheva et al., 2017, Czech Republic [35]	Case report	HNF1B-MODY	1	1	1	1	0	0	FBW, GA, mode of delivery	PPROM and spontaneous VD at 33 weeks of gestation. FBW of 2105 g.
Deng et al., 2019, China [36]	Case report	HNF1B-MODY	1	1	1	1	0	0	FBW, GA, mode of delivery	Spontaneous VD occurred at 39 + 5 weeks of gestation. FBW of 2900 g.

CS—Cesarean section; FBW—fetal birth-weight; FG—fetal genotype; FG group—initiation of insulin therapy based on fetal growth; GA—gestational age at delivery; GCK-MODY—glucokinase-Maturity Onset Diabetes of the Young; HNF1A-MODY—hepatocyte nuclear factor 1 alpha-Maturity Onset Diabetes of the Young; HNF1B-MODY—hepatocyte nuclear factor 1 beta-Maturity Onset Diabetes of the Young; LGA—large-for-gestational-age fetus; MG group—initiation of insulin therapy based on maternal capillary blood glucose values; MODY—Maturity Onset Diabetes of the Young; MODY(+)—offspring with MODY mutation; MODY(−)—offspring without MODY mutation; NH—neonatal hypoglycemia; PPROM—preterm premature rupture of membranes; SD—shoulder dystocia; SGA—small-for-gestational-age fetus; VD—vaginal delivery; n/a—not available.

## Data Availability

No new data were created or analyzed in this study. Data sharing is not applicable to this article.

## Supplementary Materials

The following supporting information can be downloaded at: https://www.mdpi.com/article/10.3390/ijms26136057/s1. Reference [14] is cited in the Supplementary Materials.
