# Supplementary material for: Pregnancy and Neonatal Outcomes in Maturity-Onset Diabetes of the Young: A Systematic Review
Source: Int J Mol Sci. 2025 Jun 24;26(13):6057. doi: 10.3390/ijms26136057 (PMC12250487; doi:10.3390/ijms26136057)
Supplement: Supplementary file 1 [file ijms-26-06057-s001.zip › ijms-3678712-supplementary.pdf]

Table S1. Characteristics of the most common variants of MODY-monogenic diabetes [14].

MODY variant	Gene mutation	Prevalence (% of MODY)	Age of onset and diagnosis	Treatment	Complications
MODY3	HNF1A	69%	teenager	1. Non-pregnant women:<br>- first-line therapy: sulfonylureas;<br>- second-line therapy: meglitinides; GLP-1 receptor agonists<br>2. Pregnant women: insulin therapy	- the earlier onset of diabetes<br>in children and young adults<br>- micro- and macrovascular complications (long-term<br>complication for pregnant<br>women)
MODY2	GCK	20%	newborn/teenager	1. Non-pregnant/ pregnant women: diet;<br>in selected cases, insulin<br>therapy	- mutation inherited: fetal<br>growth restriction<br>- mutation not inherited: large<br>for gestational age
MODY5	HNF1B	5%	newborn/teenager	1.Non-pregnant/ preg-<br>nant women: insulin therapy	- small for gestational age
MODY1	HNF4A	3%	early adulthood	1. Non-pregnant women: sulfonylure-<br>as<br>2. Pregnant wom-<br>en: insulin therapy	- fetal macrosomia<br>- neonatal hyperinsulinemic hypoglycaemia
MODY4	PDX1	<1%	newborn/early adulthood	1.Non-pregnant/ preg-<br>nant women: insulin therapy	- small for gestational age

GCK – glucokinase; GLP-1 – glucagon-like peptide-1; HNF1A – hepatocyte nuclear factor 1-alpha; HNF1B – hepatocyte nuclear factor 1-beta; HNF4A – hepatocyte nuclear factor 4-alpha; MODY – maturity-onset diabetes of the young; PDX1 – pancreatic and duodenal homeobox 1

Adapted from: Majewska, A.; Stanirowski, P.; Wielgoś, M.; Bomba-Opoń, D. Maturity-Onset Diabetes of the Young (MODY) in Pregnancy: A Review. *Curr Diabetes Rev* **2022**, *19*, doi:10.2174/1573399818666220128124043.
